# The Primacy Effect in Amnestic Mild Cognitive Impairment: Associations with Hippocampal Functional Connectivity

**DOI:** 10.3389/fnagi.2016.00244

**Published:** 2016-10-21

**Authors:** Katharina Brueggen, Elisabeth Kasper, Martin Dyrba, Davide Bruno, Nunzio Pomara, Michael Ewers, Marco Duering, Katharina Bürger, Stefan J. Teipel

**Affiliations:** ^1^German Center for Neurodegenerative Diseases (DZNE) - RostockRostock, Germany; ^2^Department of Psychosomatic Medicine, University of RostockRostock, Germany; ^3^School of Natural Sciences and Psychology, Liverpool John Moores UniversityLiverpool, UK; ^4^Nathan Kline Institute for Psychiatric ResearchOrangeburg, NY, USA; ^5^Department of Psychiatry, School of Medicine, New York UniversityNew York City, NY, USA; ^6^Institute for Stroke and Dementia Research, Klinikum der Universität München, Ludwig-Maximilians-Universität (LMU)Munich, Germany; ^7^German Center for Neurodegenerative Diseases (DZNE)Munich, Germany

**Keywords:** primacy effect, mild cognitive impairment (MCI), Alzheimer's disease (AD), functional connectivity, default mode network, hippocampus

## Abstract

**Background:** The “primacy effect,” i.e., increased memory recall for the first items of a series compared to the following items, is reduced in amnestic mild cognitive impairment (aMCI). Memory task-fMRI studies demonstrated that primacy recall is associated with higher activation of the hippocampus and temporo-parietal and frontal cortical regions in healthy subjects. Functional magnetic resonance imaging (fMRI) at resting state revealed that hippocampus functional connectivity (FC) with neocortical brain areas, including regions of the default mode network (DMN), is altered in aMCI. The present study aimed to investigate whether resting state fMRI FC between the hippocampus and cortical brain regions, especially the DMN, is associated with primacy recall performance in aMCI.

**Methods:** A number of 87 aMCI patients underwent resting state fMRI and verbal episodic memory assessment. FC between the left or right hippocampus, respectively, and all other voxels in gray matter was mapped voxel-wise and used in whole-brain regression analyses, testing whether FC values predicted delayed primacy recall score. The delayed primacy score was defined as the number of the first four words recalled on the California Verbal Learning Test. Additionally, a partial least squares (PLS) analysis was performed, using DMN regions as seeds to identify the association of their functional interactions with delayed primacy recall.

**Results:** Voxel-based analyses indicated that delayed primacy recall was mainly (positively) associated with higher FC between the left and right hippocampus. Additionally, significant associations were found for higher FC between the left hippocampus and bilateral temporal cortex, frontal cortical regions, and for higher FC between the right hippocampus and right temporal cortex, right frontal cortical regions, left medial frontal cortex and right amygdala (*p* < 0.01, uncorr.). PLS analysis revealed positive associations of delayed primacy recall with FC between regions of the DMN, including the left and right hippocampus, as well as middle cingulate cortex and thalamus (*p* < 0.04). In conclusion, in the light of decreased hippocampus function in aMCI, inter-hemispheric hippocampus FC and hippocampal FC with brain regions predominantly included in the DMN may contribute to residual primacy recall in aMCI.

## Introduction

Impaired consolidation of verbal information into long-term episodic memory is the dominant symptom in Alzheimer's disease (AD) (Carlesimo and Oscar-Berman, 1992; Pena-Casanova et al., 2012; Weintraub et al., 2012). The primacy effect relies on effective memory consolidation and refers to the phenomenon that the first items of a series are remembered better than subsequent items (Murdock, 1962). In amnestic mild cognitive impairment (aMCI), which is associated with increased risk of AD dementia (Petersen et al., 1999; Albert et al., 2011), memory consolidation is impaired and the primacy effect is reduced (Howieson et al., 2011; Cunha et al., 2012; Moser et al., 2014). A reduced recall of primacy words has been shown to predict conversion from MCI to AD (Egli et al., 2014), and to distinguish MCI patients who converted to AD from nonconverters (Cunha et al., 2012).

Primacy recall performance relies on the functionality of the hippocampal system and neocortical regions, as shown for young healthy subjects in functional magnetic resonance imaging (fMRI) studies using memory tasks (Strange et al., 2002; Talmi et al., 2005; Axmacher et al., 2009) and for elderly healthy subjects in volumetric MRI studies (Bruno et al., 2015). Especially activation of the anterior part of the hippocampal body (Strange et al., 2002) and the hippocampus in the left hemisphere (Talmi et al., 2005) were associated with the retrieval of primacy words. With regard to neocortical regions, fMRI studies in healthy subjects showed that higher activation in the (para)hippocampus and posterior fusiform gyrus was associated with higher primacy recall in a verbal memory task (Strange et al., 2002), and that higher activation in the inferior parietal lobe (including angular gyrus and supramarginal gyrus) and in the superior occipital gyrus, fusiform gyrus and cuneus were associated with higher primacy recall in a visual memory task (Sommer et al., 2006).

In aMCI, the association between primacy recall and functional brain changes has not been investigated yet. However, a volumetric MRI study in aMCI showed that the primacy effect was associated with volume of the parieto-temporal lobe, including the supramarginal gyrus in inferior parietal regions, as well as the middle and inferior temporal gyrus (Kasper et al., 2016). Given previous findings on the central role of hippocampus activation in primacy recall in healthy adults and the fact that the hippocampus is impaired in aMCI (e.g., Apostolova et al., 2010), we aimed to test whether functional connectivity of the hippocampus to other cortical areas typically involved in memory is associated with residual primacy recall in aMCI. Functionally connected regions that have been associated with episodic memory include regions of the default mode network (DMN; i.e., ventral parietal, posterior cingulate, medial prefrontal, and hippocampal regions; Huijbers et al., 2011), as well as basal ganglia, cerebellum, temporal lobe regions (Bai et al., 2009), and parietal cortical regions (Kim, 2011).

Thus, in the present study, we aimed at investigating the association of hippocampal functional connectivity (FC) at resting state with delayed recall of primacy words in patients with aMCI. We hypothesized that higher resting state hippocampal FC (especially FC of the anterior and left hippocampus) with frontal, temporal and parietal cortical regions of the brain, particularly areas belonging to the DMN, would be associated with higher delayed primacy recall. To address this issue, we used linear regression analyses and a partial least squares (PLS) analysis, which allowed the simultaneous assessment of functional connectivity of these regions in a multivariate framework (Krishnan et al., 2011).

## Methods

### Subjects

The study sample consisted of 87 individuals (44 female; mean age 74 years, *SD*: 5.6, range: 61–87) diagnosed with aMCI according to the Peterson criteria (including subjective memory complaints, objectively deviant scores on memory tests, intact general mental status, normal daily functioning, and absence of dementia; Petersen et al., 1999). *N* = 33 patients were classified as single domain aMCI subtype (i.e., exhibiting an exclusive memory impairment); *n* = 54 patients were classified as multiple domain aMCI subtype (i.e., exhibiting an impairment in the memory domain as well as other cognitive domains; Petersen et al., 2001; Petersen, 2004). For a detailed neuropsychological characterization, see Supplementary Table 1. The sample was recruited for an intervention study at the University Hospital Munich, Germany. Ethical approval was given by the local ethics committee of the Faculty of Medicine at the Ludwig-Maximilian University in Munich, Germany. All subjects gave written informed consent in accordance with the Declaration of Helsinki. Based on the German education system, the subjects' education levels were converted to a categorical scale ranging from 1 (i.e., no educational qualification) to 5 (i.e., university degree), resulting in a frequency distribution of education category 1: *n* = 23, category 2: *n* = 22, category 3: *n* = 19, category 4: *n* = 23. The mean MMSE score was 27 (*SD*: 1.7; range: 22–30).

### Neuropsychological assessment

Verbal episodic memory was assessed using the German version of the California Verbal Learning Test (CVLT) (Niemann et al., 2008), a standardized test of verbal memory, consisting of a word list A and a distractor word list B. Each list contained 16 words, belonging to one of four semantic categories. List A was read to the participants five times, followed by immediate free recall after each learning trial. After the fifth trial, the distractor word list B was read to the participants, followed by immediate free recall of list B. After this, participants were asked to recall all words from list A. After a 20-min delay, participants were asked to recall the words again (“delayed recall”). The number of correctly recalled words from the first four words of list A at delayed recall was defined as “delayed primacy recall” (La Rue et al., 2008; Bruno et al., 2013; Egli et al., 2014). Additionally, “delayed total recall” was defined as the number of correctly recalled words out of all 16 words from list A at delayed recall.

### MR data acquisition

Subjects were scanned using a 3T Siemens Magnetom Verio Scanner (Siemens, Erlangen, Germany) with a 12-channel head coil. The T1-weighted high-resolution structural MRI volumes were scanned with a 3D magnetization-prepared rapid gradient-echo MPRAGE sequence (TE = 3.06 ms, repetition time (TR) = 2100 ms, inversion time (TI) = 900 ms, bandwidth = 230 Hz per pixel, matrix: 240 × 256 × 160, isotropic voxel size: 1.0 mm). The functional images were acquired during resting state by echo-planar imaging (EPI) [scan time = 6 min, repetition time (TR) = 3000 ms, echo time (TE) = 30 ms, flip angle = 80°, field of view (FOV) = 192 × 192 × 112 mm]. A number of 28 slices was acquired (interleaved, ascending), with 4 mm thickness and 0.4 mm gap, resulting in 3 × 3 × 4.4 mm voxel resolution and 120 volumes.

### MR data preprocessing

Segmentation and normalization of the anatomical T1-weighted MPRAGE scans was performed using VBM8 (Gaser et al., 1999). The anatomical images were coregistered to the fMRI scans for each subject. The coregistered structural MPRAGE scans were segmented into gray matter (GM), white matter (WM), and cerebrospinal fluid (CSF). Subsequently, the scans were normalized onto the default IXI template provided by VBM8, using DARTEL (Ashburner, 2007). The resulting warping parameters were used to transform each subject's fMRI volumes to the MNI reference space.

The preprocessing of the fMRI data was carried out using Data Processing Assistant for Resting-State MRI Advanced (DPARSFA, version 3.1, http://rfmri.org/DPARSF; Yan and Zang, 2010), which is based on Statistical Parametric Mapping (SPM8; http://www.fil.ion.ucl.ac.uk/spm). Preprocessing included removal of the first six volumes of each fMRI scan, slice timing correction to the temporal middle, head motion correction to the mean volume, nuisance covariate regression with 12 motion parameters (translation, rotation, and derivatives), temporal bandpass filtering (0.01–0.1 Hz), normalization using the deformation fields previously obtained for the anatomical images to project the fMRI, reslicing to an isotropic voxel size of 1.5 mm, and spatial smoothing with a 4 mm isotropic full-width-at-half-maximum (FWHM) Gaussian kernel.

The left and right hippocampus were manually delineated by a trained expert using the GM segment obtained from the VBM8 IXI template (Grothe et al., 2012). Using DPARSFA, averaged time courses were obtained using the left or right hippocampus as seed, respectively, and Pearson-Moment correlation analysis was performed between the BOLD time course within the hippocampus (averaged across all voxels) and each voxel in the brain. This resulted in FC coefficient maps for each subject, which were converted into z-maps by Fisher's r-to-z transformation to be used in subsequent statistical analyses. The GM segment of the IXI template was thresholded at *p* < 0.3 to define the GM mask, which was applied to the FC maps to restrict the analyses to areas within the GM only. One-sample *t*-tests were then used to threshold the resulting FC maps at *p* < 0.001 (uncorr.) to obtain binary inclusive masks that were used in all following regression analyses, restricting results to functionally connected voxels.

### Statistical analysis

For comparing the number of correctly recalled primacy words at delayed recall to the number of correctly recalled words from the rest of the list, proportions were calculated and compared by means of a paired samples *t*-test.

Multiple linear regression analyses were carried out using SPM8 (Wellcome Department of Imaging Neuroscience, London, UK; Friston et al., 2007) to regress delayed primacy recall voxel-wise on Fischer z-transformed FC coefficients within the FC map of voxels functionally connected to the hippocampus, adjusted for age, gender and education. Separate analyses were conducted for the left and right hemispheres. All regression analyses were repeated with a subset of *n* = 53 subjects, encompassing only subjects without floor effects (i.e., delayed primacy recall ≥ 1). Moreover, regression analyses were repeated additionally controlling for delayed total recall, and additionally controlling for left or right hippocampal volume (using all subjects). Lastly, a median split was performed based on delayed total recall, separating the sample into a high and a low performing group, and regression analyses were repeated for the two subgroups. Only positive associations were tested. For all regression analyses, a cluster threshold of ≥ 20 voxels was applied.

Multivariate partial least squares (PLS) analysis was performed in Matlab (McIntosh et al., 1996; McIntosh and Lobaugh, 2004) to assess the covariance of delayed primacy recall with patterns of FC between nine seed regions of the DMN (including regions within posterior cingulate/precuneus, middle cingulate cortex, angular gyrus, hippocampus, medial orbitofrontal cortex, medial prefrontal cortex, superior frontal cortex, and thalamus, as defined in a FC-based atlas that was warped to MNI space (Shirer et al., 2011; Supplementary Figure 1). In general, a PLS analysis is based on a linear multivariate model. Taking their structure into account, it simultaneously decomposes matrices and constructs so-called orthogonal latent variables (LV) to model the relationship between two data matrices X and Y. Compared to multiple linear regression, it has the advantage of being able to handle datasets with a large number of predictors and high collinearity (Wold et al., 2001). In the present study, the PLS analysis identified a LV that best captured the covariance between the FC coefficients for each pair of seed regions (contained in matrix X) and the delayed primacy recall scores (contained in matrix Y; Abdi, 2010). Before the correlation coefficients entered the PLS analysis, the influence of age, gender and education was linearly regressed out (for formulae see Dyrba et al., 2015a,b). For each value in matrix X, a salience was calculated, indicating how strongly it contributed to the LV. Bootstrap-ratios (BSR) using 1000 iterations were calculated for each salience to determine its reliability, with BSR ≥ 2.0 indicating reliability, as described in Abdi and Williams (2013). As saliences were identified in a single analytic step, no correction for multiple comparisons was needed (McIntosh et al., 1996). The statistical significance of the LV was determined using permutation testing with 1000 iterations.

## Results

### Behavioral results

The proportion of correctly recalled primacy words at delayed recall (mean: 0.32, *SD*: 0.33) was significantly higher than the proportion of correctly recalled words from the rest of the list (mean: 0.25, *SD*: 0.24; paired samples *t*-test, *p* < 0.01). *N* = 34 subjects recalled no primacy words (reaching a floor effect).

### Functional connectivity of the hippocampus

**Figure 1:**
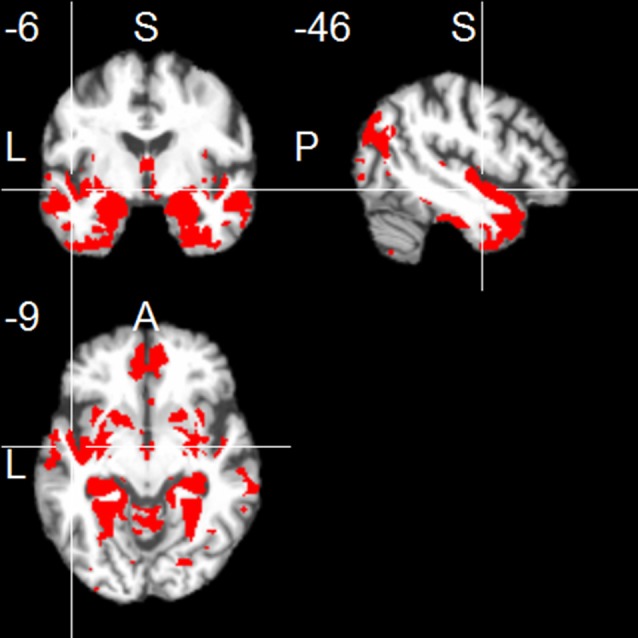
**Positive functional connectivity of the left hippocampus**. Thresholded at *p* < 0.001, uncorrected for multiple comparisons, cluster size ≥ 20 voxels.

**Figure 2:**
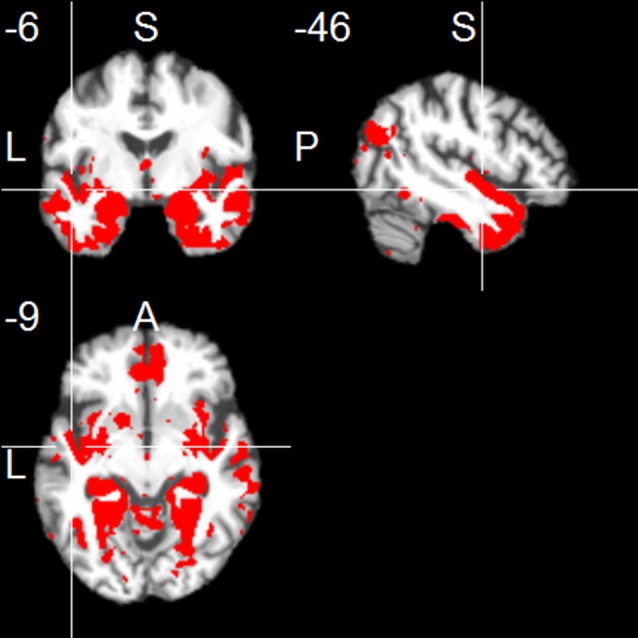
**Positive functional connectivity of the right hippocampus**. Thresholded at *p* < 0.001, uncorrected for multiple comparisons, cluster size ≥ 20 voxels.

The left hippocampus showed functional connections with clusters in frontal regions (mainly medial orbitofronal cortex), temporal regions (including inferior, middle and superior temporal regions, contralateral hippocampus, parahippocampal region, temporal pole, and fusiform gyrus), and parietal regions (including a parieto-occipital region, posterior angular gyrus, and medial regions including PCC and precuneus; one-sample *t*-test, *p* < 0.001, uncorr.; Figure 1). The right hippocampus showed positive FC with similar cortical regions (one-sample *t*-test, *p* < 0.001, uncorr.), most prominently with temporal regions (including inferior, middle and superior temporal gyrus, temporal pole, fusiform gyrus, insula, and the contralateral hippocampus), thalamus, frontal regions (including superior frontal gyrus, and medial orbitofrontal gyrus), parietal regions (including PCC/precuneus, and posterior angular gyrus), and cerebellum (Figure 2).

### Association of delayed primacy recall and hippocampal functional connectivity

#### Linear regression analyses

**Figure 3:**
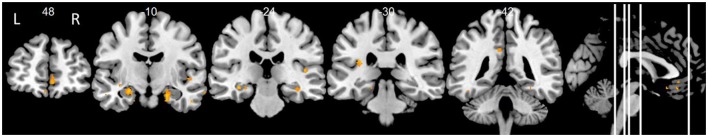
**Positive associations of delayed primacy recall and left hippocampal functional connectivity**. Controlled for age, gender, and education. Maps were thresholded at significance level *p* < 0.01 (uncorrected for multiple comparisons), cluster size ≥ 20 voxels.

We found positive correlations of delayed primacy recall and FC between the *left hippocampus* and clusters in the right hippocampus, left superior temporal pole, left insula, left posterior cingulate cortex, left putamen, left superior frontal gyrus, bilateral medial orbitofrontal cortex, right superior temporal cortex, right middle temporal gyrus, right fusiform gyrus, and right amygdala (*p* < 0.01, uncorr.; Figure 3, Table 1). At *p* < 0.001, FC with the right hippocampus remained significant. Leaving out floor effects resulted in additional clusters in medial parietal areas (including left precuneus) and occipital regions (including left calcarine and left cuneus; *p* < 0.01, uncorr.; Supplementary Table 2). Correction for delayed total recall (with all subjects) resulted in additional clusters in the inferior temporal and occipital cortex, and the cerebellum (Supplementary Table 4). Results were consistent when controlling for left hippocampal volume, except for a few clusters no longer significant at *p* < 0.01, located in the frontal cortex (MNI coordinates: *x* = −22, *y* = 23, *z* = 45; *x* = −6, *y* = 42, *z* = −17), posterior cingulate cortex (*x* = −8, *y* = −40, *z* = 33), putamen (*x* = −21, *y* = 14, *z* = −11), temporal cortex (*x* = −38, *y* = −13, *z* = −11; *x* = 69, *y* = −16, *z* = −14), and fusiform gyrus (*x* = 45, *y* = −20, *z* = −29).

**Table 1:** **Positive associations of delayed primacy recall and FC of left and right hippocampus (controlled for age, gender and education; ***p*** < 0.01, uncorr., cluster size ≥ 20 voxels)**.

**Brain region**		**Peak MNI coordinates of local maxima (*x y z*) (mm)**	**Peak T score**	**Cluster size (voxel count)**
**LEFT HIPPOCAMPAL FC**				
R/L	Medial orbitofrontal cortex	8 34 −14	4.42	70
		−2 34 −14	3.14	
		3 51 −8	3.37	59
		6 48 0	2.59	
		−6 42 −17	3.17	29
L	Hippocampus	−34 −22 −12	4.02	175
		−24 −10 −17	3.66	
		−27 −18 −17	2.99	
R	Hippocampus^†^	21 −10 −23	3.81	290
		26 −16 −14	3.23	
		34 −19 −15	3.11	
L	Superior temporal pole	−40 15 −24	3.75	25
L	Insula	−33 −31 21	3.66	46
R	Superior temporal cortex	48 −7 −3	3.55	28
		−38 −13 −11	3.25	30
R	Fusiform gyrus	45 −20 −29	3.54	24
L	Superior frontal gyrus	−22 23 45	3.53	20
R	Amygdala	20 −3 −12	3.38	25
L	Putamen	−21 14 −11	3.30	23
L	Posterior cingulate cortex	−8 −40 33	3.08	21
R	Middle temporal gyrus	69 −16 −14	2.70	22
**RIGHT HIPPOCAMPAL FC**				
R	Middle temporal cortex	66 −10 −18	4.53	33
L	Hippocampus^†^	−27 −19 −15	4.26	370
		−21 −12 −18	3.56	
		−36 −27 −24	3.19	
R	Medial orbitofrontal cortex	3 51 −8	3.77	85
	Anterior cingulate cortex	9 36 −5	2.99	
	Medial superior frontal cortex	4 48 1	2.75	
R	Inferior temporal cortex	56 −16 −29	3.60	43
L	Thalamus/Hippocampus	−15 −31 1	3.32	20
		−22 −37 1	2.85	
R	Amygdala	20 −1 −14	3.21	24
L	Medial frontal cortex	3 30 −17	3.04	44

† *Cluster remained significant at p < 0.001*.

**Figure 4:**
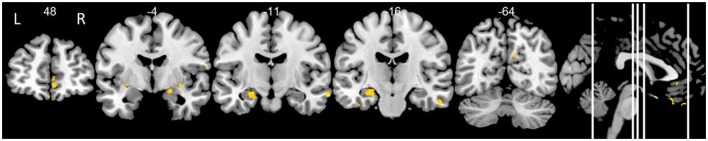
**Positive correlations of delayed primacy recall and right hippocampus**. Controlled for age, gender, education. Maps were thresholded at significance level *p* < 0.01 (uncorrected for multiple comparisons), cluster size ≥ 20 voxels.

Using the *right hippocampus* as seed, delayed primacy recall and FC correlated in frontal, temporal and parietal regions (*p* < 0.01, uncorr.), corrected for age, gender and education, comparable to FC of the left hippocampus (Figure 4, Table 1). At *p* < 0.001, FC with the left hippocampus remained significant. Leaving out subjects with floor effects, delayed primacy recall correlated with FC of the right hippocampus and bilateral frontal and temporal regions, as well as right posterior cingulate cortex (*p* < 0.01, uncorr.; Supplementary Table 3). Correction for delayed total recall resulted in additional clusters in insula/putamen, Heschl's gyrus, posterior cingulate cortex, temporal pole and fusiform gyrus (Supplementary Table 5). Results were consistent when controlling for right hippocampal volume. A comparison of two subgroups, categorized based on their delayed total recall performance, indicated that associations between inter-hemispheric FC and delayed primacy recall were especially pronounced in the group with higher memory performance (data not shown).

#### Partial least squares analysis

**Figure 5:**
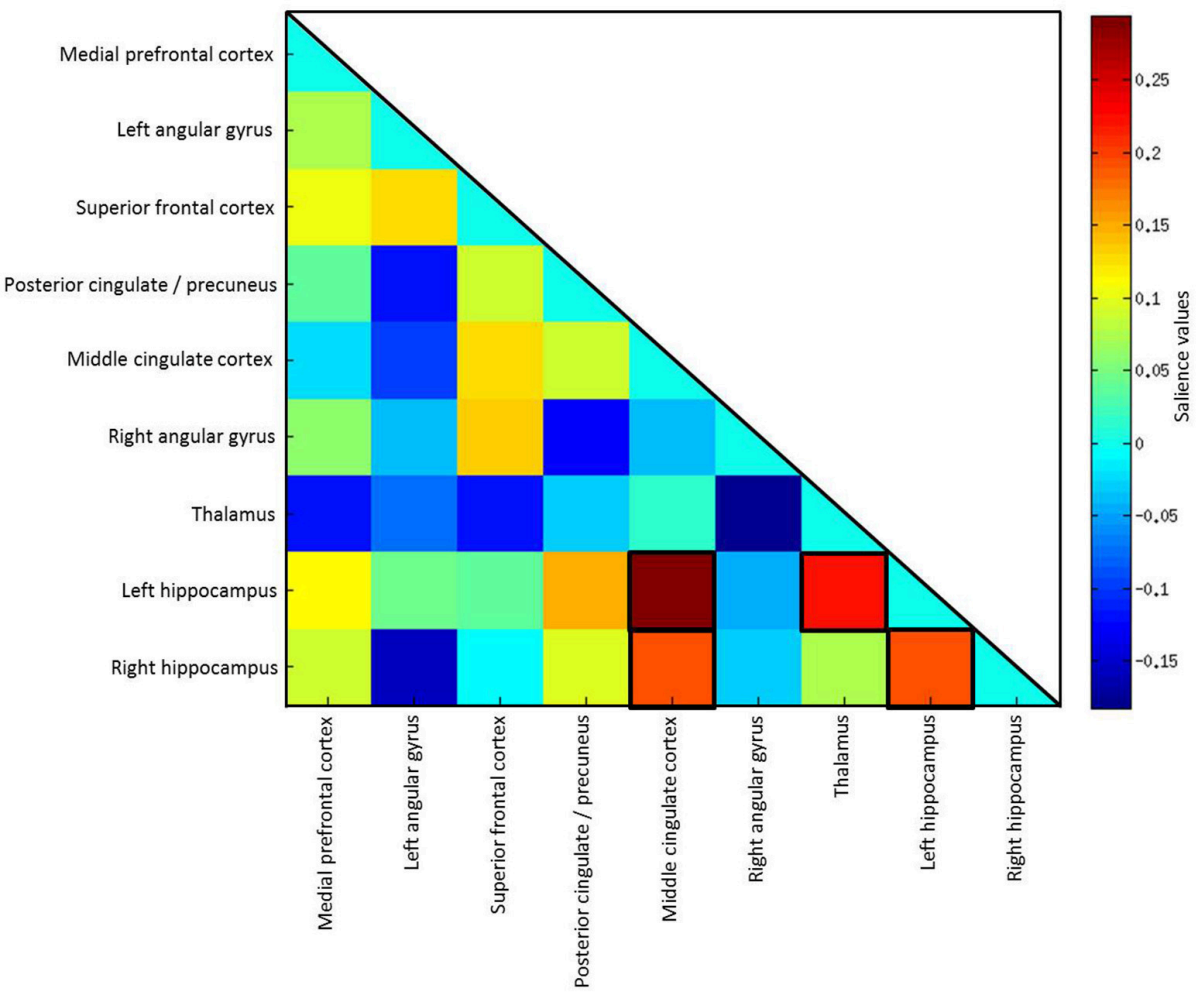
**Salience values of the latent variable**. The highest 10% of salience values are highlighted by black frames.

Effects of the covariates age, gender and education were regressed out before coefficients entered the analysis. PLS analysis using all subjects (*n* = 87) did not reach statistical significance (*p* = 0.262). Including only subjects who did not show floor effects (*n* = 53), results of the PLS analysis revealed positive correlations of FC coefficients with delayed primacy recall. The 10% highest LV saliences were found for FC between the middle cingulate cortex (bilateral) and the left hippocampus (BSR = 4.51), thalamus (bilateral) and hippocampus (left) (BSR = 2.69), left and right hippocampus (BSR = 2.67), as well as middle cingulate cortex (bilateral) and the right hippocampus (BSR = 2.69; *p* < 0.04; Figure 5).

## Discussion

In the present study, verbal memory consolidation, as indicated by delayed primacy recall, was mainly (positively) associated with inter-hemispheric FC between the hippocampi, as well as hippocampal FC with the temporal, frontal and middle cingulate cortex and the thalamus.

The association between resting state FC and primacy recall has not been investigated before. However, studies have assessed the association between resting state FC and episodic memory performance. Comparable to our findings, these studies showed significant positive associations of episodic memory with FC in medial temporal regions (Wang et al., 2012) and with FC between the hippocampus and regions of the DMN in HC (Salami et al., 2014) and in MCI (Wang et al., 2010; Bai et al., 2011; Zhou et al., 2015).

The brain regions involved in episodic memory are numerous, as shown by task-fMRI studies in HC (Cabeza, 2004; Dickerson et al., 2007; Axmacher et al., 2009; Spaniol et al., 2009; for a meta-analyis, see Kim, 2011). Only one task-fMRI study investigated regions involved in the primacy effect for verbal information, showing a significant association with activation in the (para)hippocampus and posterior fusiform gyrus (Strange et al., 2002). A meta-analysis of task-fMRI studies investigating verbal episodic memory consolidation (regardless of serial position effects) in HC showed the most consistent activations in the temporal pole, fusiform gyrus, left inferior frontal cortex, hippocampal formation, premotor cortex and posterior cingulate cortex (Kim, 2011). In AD, the functional networks spanning these regions are impaired. Especially the FC of the hippocampus with PCC/precuneus (Wang et al., 2010; Kim et al., 2013; Sohn et al., 2014) and other parts of the DMN (Greicius et al., 2004; Vincent et al., 2006; Wang et al., 2006, 2010; Allen et al., 2007; Sorg et al., 2007; Zhang et al., 2009) are among the first to be disrupted. In the present study, we specifically investigated the role this network plays in the residual consolidation at the predementia stage aMCI. Our results suggest that mainly inter-hemispheric hippocampal FC is associated with the remaining memory consolidation.

On the other hand, some studies suggested an age-related increase in FC between the left and right hippocampus to be associated with episodic memory deficits in healthy aging (Salami et al., 2014) and MCI/AD (Pasquini et al., 2015). The increase in FC may be due to the loss of inhibitory cortical input resulting from decreased hippocampal-cortical FC, and the functional isolation of the hippocampus may result in mnemonic processing deficits (Salami et al., 2014). It might be possible that the inter-hippocampal FC found in the present study is upregulated, compensating for the decreased hippocampal function to maintain memory consolidation. This was supported by a particularly strong association between inter-hippocampal FC and delayed primacy recall in a subgroup with less memory impairment (categorized by a median split) in our study. Future studies using a HC group might further investigate this aspect.

Interestingly, in the present study, we found that delayed primacy recall did not strongly correlate with FC between the hippocampus and the inferior parietal cortex. This was unexpected for a number of reasons. Firstly, the inferior parietal cortex is part of the attention network, and it is functionally connected to the hippocampus at resting state (Cabeza et al., 2008; Daselaar et al., 2013). Secondly, it has been shown to be activated during successful retrieval (Vincent et al., 2006), and BOLD activity in the inferior parietal cortex and angular gyrus during encoding was reported to be associated with primacy recall (Sommer et al., 2006). Our findings can be explained by the fact that the hippocampal FC to parietal cortical regions was restricted to posterior parts of the inferior parietal cortex, including parieto-occipital regions and the posterior part of the angular gyrus. The region positioned anterior to this, i.e., the supramarginal gyrus, was not significantly functionally connected with the hippocampus. A volumetric study using the same study sample showed that volume decrease of the bilateral supramarginal gyri was significantly associated with impaired delayed primacy recall (Kasper et al., 2016). It is possible that the early structural damage of both the supramarginal gyrus and the hippocampus underlies the reduced FC between these regions.

In addition, results of previous studies indicated a tendency for a left lateralization regarding the role of the hippocampus in verbal memory (Richardson et al., 2003; Talmi et al., 2005; Travis et al., 2014) and autobiographical memory retrieval (Maguire et al., 2001). In the present study, associations between delayed primacy recall and FC of the hippocampus with the superior temporal cortex, temporal pole, insula, fusiform gyrus, putamen, and posterior cingulate cortex were exclusively found for the left hippocampus, and were not significant for the right hippocampus. This could not be explained by an overall increase in FC of the left hippocampus, as the FC maps of the left and right hippocampus with whole-brain voxels showed correlations of the same magnitude with similar regions. Moreover, when controlling for volume of the left hippocampus, most of the above-mentioned associations that were present exclusively for the left hippocampus no longer reached statistical significance, so that the associations between delayed primacy recall and FC of the left and right hippocampus became more similar. As the left hippocampus atrophies earlier than the right hippocampus in aMCI and AD (Janke et al., 2001; Thompson et al., 2003), increased recruitment of remote brain regions reflected in increased FC might serve to maintain verbal memory consolidation.

We used a PLS analysis to identify associations of FC patterns between regions of the DMN with delayed primacy recall. This approach allowed for the simultaneous assessment of functional connectivity between several seeds. Being able to handle a large number of predictors compared to the number of observations, a PLS approach is particularly useful for analyzing imaging data, which is typically highly collinear (Abdi, 2010; Krishnan et al., 2011). The PLS analysis resulted in a latent variable representing a pattern of FC reliably associated with delayed primacy recall. The highest saliences of this variable included FC between the bilateral hippocampi, lending support to the results of the linear regression analyses, which also indicated inter-hippocampal FC to play a major role in verbal memory consolidation. Additionally, high saliences were found for hippocampal FC with the middle cingulate cortex and thalamus, which might suggest a compensatory recruitment of these regions.

A limitation of this study is the lack of a HC group. However, the present study did not aim at replicating the well-established fact that verbal memory consolidation is impaired in aMCI. Instead, it intended to assess how variation in verbal memory consolidation relates to hippocampal FC at the prodromal AD stage. A group comparison of FC would have added information on altered resting state networks in aMCI in more detail; however, a large number of studies have already investigated FC alterations in MCI patients, compared with controls.

Secondly, a relatively low statistical significance threshold was applied for the regression analyses, compared to volumetric studies. Resting state fMRI analyses capture spontaneous brain function within functional networks that are not static over time (Allen et al., 2012), and can be susceptible to variability (Cole et al., 2010). Moreover, inter-session test-retest reliability of resting state networks may be reduced in MCI compared to HC (Blautzik et al., 2013). Yet, the main findings in this study exhibited large cluster sizes, thereby reducing the probability of false positives. In addition, using a scan time that lasted long enough to produce reliable results (van Dijk et al., 2010) and a sufficiently large sample size increased the robustness of the effects. Furthermore, the results of the regression analyses were supported by a multivariate PLS analysis, which is particularly suited for handling imaging data with multicollinearity (Abdi, 2010; Krishnan et al., 2011).

Our results expand upon previous research that showed hippocampal volume to be associated with delayed primacy recall in HC (Bruno et al., 2015), and hippocampal volume and function to be reduced in aMCI (e.g., Jack et al., 1999; Chetelat and Baron, 2003; Chetelat et al., 2005; Devanand et al., 2012). Future longitudinal studies are needed to investigate the role that the correlation between FC and memory consolidation may play in the prediction of conversion to dementia. Earlier studies on serial position effects in MCI suggested that primacy recall may predict conversion to AD, especially at early MCI stages (Cunha et al., 2012; Egli et al., 2014, 2015).

In conclusion, inter-hippocampal FC and hippocampal FC with temporal, frontal and middle cingulate cortex and the thalamus may account for residual verbal memory consolidation in aMCI. Assessing FC between these regions could provide important information during an early stage of AD and may aid the stratification of subjects in clinical trials.

## Author contributions

KBr performed analyses and interpretation of the data and drafted and revised the manuscript. EK substantially contributed to the design of the work and the analysis, and critically revised the manuscript. MDy contributed to the statistical design, interpretation of the results and revised the work for intellectual content and format. DB provided background knowledge for this study, contributed to the design and interpretation and revised the manuscript. NP contributed to the study design and revised the work. ME contributed to the acquisition of the data and interpretation of the results and revised the manuscript. MDu also contributed to the acquisition and revised the manuscript for content and clarity. KBü contributed to the acquisition and study design, provided feedback and revised the manuscript. ST was involved in all stages of the work, contributing to the study design, research question and analyses, and critically revising the manuscript. All authors agreed for the manuscript to be published and to be accountable for the work in ensuring that the accuracy and integrity are given or, if necessary, investigated and resolved.

### Conflict of interest statement

The authors declare that the research was conducted in the absence of any commercial or financial relationships that could be construed as a potential conflict of interest.
