# Supplementary material for: The Primacy Effect in Amnestic Mild Cognitive Impairment: Associations with Hippocampal Functional Connectivity
Source: Front Aging Neurosci. 2016 Oct 21;8:244. doi: 10.3389/fnagi.2016.00244 (PMC5073133; doi:10.3389/fnagi.2016.00244)
Supplement: Supplementary file 1 [file Table1.DOCX]

Supplementary Material

The Primacy Effect in amnestic Mild Cognitive Impairment: Associations with hippocampal functional connectivity

Brueggen K*, Kasper E, Dyrba M, Bruno D, Pomara N, Ewers M, Duering M, Buerger K, Teipel S

*** Correspondence:** Katharina Brüggen, [katharina.brueggen@dzne.de](mailto:katharina.brueggen@dzne.de)

## Supplementary Tables

**Supplementary Table 1. Neuropsychological characterization**

|  |  | **Total sample**  **(n = 87)** | | | **aMCI single domain**  **(n = 33)** | | | **aMCI multiple domain**  **(n = 54)** | | |
| --- | --- | --- | --- | --- | --- | --- | --- | --- | --- | --- |
| **Cognitive domain** | **CERAD subtest** | **Mean** | **SD** | **Range** | **Mean** | **SD** | **Range** | **Mean** | **SD** | **Range** |
| **Attention** | Trail MakingTest A | -0.6 | 1.0 | -3.2 − 1.9 | -0.1 | 0.9 | -1.8 – 1.9 | -0.9 | 0.9 | -3.2 – 1.7 |
| **Memory** | Word list learning | -12 | 1.0 | -4.2 − 0.6 | -1.8 | 0.9 | -4.0 – 0.6 | -2.1 | 1.0 | -4.2 – 0.2 |
|  | Word list recall | -1.5 | 0.9 | -4.2 − 0.3 | -1.3 | 0.8 | -2.9 – 0.3 | -1.6 | 1.0 | -4.2 – 0.0 |
|  | Word list recognition | -1.5 | 1.4 | -5.3 − 0.9 | -1.5 | 1.7 | -5.3 – 0.9 | -1.4 | 1.2 | -5.0 – 0.7 |
|  | Constructional praxis recall | -1.0 | 1.5 | -3.4 − 2.5 | -0.2 | 1.6 | -3.0 – 2.5 | -1.4 | 1.2 | -3.4 – 1.3 |
| **Executive functions** | Trail MakingTest B | -0.5 | 1.1 | -2.5 − 2.9 | -0.1 | 1.0 | -2.4 – 1.5 | -1.0 | 1.1 | -2.5 – 2.9 |
|  | Phonemic fluency | 0.2 | 1.2 | -3.4 − 2.8 | 0.8 | 1.2 | -2.6 – 2.8 | -0.2 | 1.1 | -3.4 – 2.0 |
| **Visuospatial skills** | Constructional praxis | 0.1 | 1.2 | -2.5 − 1.7 | 0.4 | 1.1 | -2.4 – 1.7 | -0.1 | 1.2 | -2.5 – 1.6 |
| **Semantic memory/ language** | Semantic fluency | -0.9 | 1.1 | -3.7 − 2.5 | -0.1 | 0.9 | -1.4 – 2.5 | -1.3 | 1.0 | -3.7 – 0.9 |
|  | Boston Naming Test | -0.3 | 1.3 | -3.6 − 1.6 | 0.3 | 0.9 | -1.4 – 1.6 | -0.7 | 1.3 | -3.6 – 1.6 |

Abbreviations: aMCI: amnestic mild cognitive impairment; SD*:* standard deviation; CERAD: Consortium to Establish a Registry for Alzheimer’s Disease

Supplementary Table 2. Positive associations of delayed primacy recall and left hippocampal FC without floor effects (*n* = 53; controlled for age, gender, education; p < 0.01, uncorr., cluster size ≥ 20 voxels)

| Brain region | | Peak MNI coordinates  (x y z) (mm) | Peak T-score | Cluster size (voxel count) |
| --- | --- | --- | --- | --- |
| R | Inferior temporal gyrus | 46 -18 -30 | 5.32 | 60 |
| L | Inferior temporal gyrus | -40 15 -24  -52 -27 -26 | 4.22  3.92 | 24  58 |
| L | Precuneus | -9 -60 19  -4 -39 48 | 3.73  3.24 | 59  28 |
| L | Calcarine | -27 -66 6 | 3.69 | 31 |
| R | Hippocampus | 22 -12 -21  28 -19 -18  34 -21 -12 | 3.24 | 160 |
| R | Medial orbitofrontal cortex | 3 50 -5 | 3.22 | 43 |
| R | Fusiform gyrus | 30 -1 -42 | 3.21 | 25 |
| R | Superior temporal gyrus | 50 -7 -5 | 3.13 | 24 |
| L | Putamen | -16 8 -11 | 3.06 | 26 |

Supplementary Table 3. Positive associations of delayed primacy recall and right hippocampal FC without floor effects (*n* = 53; controlled for age, gender, education; p < 0.01, uncorr., cluster size ≥ 20 voxels)

| Brain region | | Peak MNI coordinates  (x y z) (mm) | Peak T-score | Cluster size (voxel count) |
| --- | --- | --- | --- | --- |
| R | Lingual cortex | 21 -51 -2 | 3.57 | 23 |
| R | Middle temporal gyrus | 66 -15 -17 | 3.56 | 20 |
| L | Inferior temporal gyrus | -46 -19 -29 | 3.47 | 96 |
| L | Hippocampus | -27 -18 -17  -20 -12 -18  -36 -28 -15 | 3.44  3.25  2.61 | 200 |
| R | Parahippocampal gyrus | 20 -4 -38 | 3.33 | 23 |
| R | Temporal pole | 45 16 -33 | 3.28 | 26 |
| R | Medial orbitofrontal cortex | 3 51 -9  4 48 1 | 3.13  2.84 | 47 |
| R | Posterior cingulate cortex | 9 -49 31 | 2.88 | 30 |

Supplementary Table 4. Positive associations of delayed primacy recall and left hippocampal FC (controlled for delayed total recall, age, gender, education; p < 0.01, cluster size ≥ 20 voxels)

| Brain region | | Peak MNI coordinates  (x y z) (mm) | Peak T-score | Cluster size (voxel count) |
| --- | --- | --- | --- | --- |
| L | Hippocampus | -36 -24 -12  -30 -18 -18  -26 -25 -14 | 5.29  3.56  3.24 | 227 |
| R | Inferior temporal gyrus | 48 -20 -29  54 -12 -29  34 3 -41  58 -22 -24 | 4.44  3.93  3.80  2.99 | 36  32  57  29 |
| L | Parahippocampal gyrus | -18 -31 -21 | 4.29 | 36 |
| R | Superior orbital cortex | 16 51 -23 | 4.07 | 35 |
| L | Medial orbitofrontal cortex | -6 48 -17 | 4.05 | 41 |
| L | Cerebellum | 2 -57 -6 | 4.04 | 50 |
| R | Fusiform gyrus | 27 -48 -14  34 -4 -39  44 -48 -23 | 3.85  3.20  2.76 | 76  30  20 |
| L | Superior orbital cortex | -10 54 -21 | 3.73 | 34 |
| R | Hippocampus | 34 -22 -12  22 -12 -21  16 -4 -14  27 -40 0 | 3.71  3.42  3.62  3.43 | 195  39  45 |
| L | Insula | -32 -28 19  -36 -12 -8 | 3.65  3.21 | 40  36 |
| L | Inferior temporal cortex | -54 -27 -27  -56 -33 -21 | 3.59  3.09 | 54 |
| L | Middle temporal gyrus | -52 -7 -21  -51 0 -18 | 3.44  3.26 | 60 |
| L | Cuneus | -12 -73 4 | 3.30 | 26 |
| R | Lingual gyrus | 21 -49 -2 | 3.27 | 31 |
| R | Cuneus | 16 -64 16 | 3.19 | 28 |

Supplementary Table 5. Positive associations of delayed primacy recall and right hippocampal FC (controlled for delayed total recall, age, gender, education; p < 0.01, uncorr., cluster size ≥ 20 voxels)

| Brain region | | Peak MNI coordinates  (x y z) (mm) | Peak T score | Cluster size (voxel count) |
| --- | --- | --- | --- | --- |
| L | Insula / Putamen | -36 -12 -8  -32 -4 -9  -26 3 -6 | 5.03  3.28  2.44 | 188 |
| L | Hippocampus | -36 -22 -14  -24 -13 -21  -27 -16 -12 | 4.69  3.47  2.75 | 261 |
| L | Medial orbitofrontal cortex | 0 44 -29 | 4.58 | 20 |
|  |  | -3 48 -20  -8 36 -17 | 4.16  2.87 | 50 |
|  |  | -12 21 -17 | 3.40 | 27 |
| R | Medial orbitofrontal cortex | 6 33 -15  6 40 -18 | 3.70  3.41 | 88 |
|  |  | 4 32 -27 | 3.06 | 44 |
| L | Superior orbital cortex | -12 52 -24 | 4.38 | 27 |
| R | Heschl’s gyrus | 45 -24 7 | 4.06 | 23 |
| R | Anterior cingulate cortex | 2 38 -5 | 3.95 | 40 |
| R | Inferior temporal gyrus | 56 -16 -27  52 -24 -26  50 -33 -26 | 3.92  3.53  3.45 | 160 |
|  |  | 46 -57 -23 | 3.03 | 21 |
| L | Inferior temporal cortex | -42 -19 -32 | 2.85 | 28 |
| L | Lingual cortex | -14 -67 3 | 3.56 | 23 |
| L | Middle temporal gyrus | -54 -6 -21 | 3.06 | 20 |
| R | Middle temporal gyrus | 52 -19 -15 | 3.53 | 20 |
| L | Posterior cingulate cortex | -4 -42 30 | 3.49 | 20 |
| R | Paracingulate cortex | 4 50 -2 | 3.26 | 38 |
| R | Sulcus calcarine | 16 -64 18 | 3.23 | 24 |
| R | Superior temporal pole | 39 3 -23 | 3.08 | 29 |
| R | Fusiform gyrus | 28 -40 -15 | 3.02 | 48 |
