# Supplementary material for: The Primacy Effect in Amnestic Mild Cognitive Impairment: Associations with Hippocampal Functional Connectivity
Source: Front Aging Neurosci. 2016 Oct 21;8:244. doi: 10.3389/fnagi.2016.00244 (PMC5073133; doi:10.3389/fnagi.2016.00244)
Supplement: Supplementary file 2 [file Image1.PDF]

Supplementary Material

The Primacy Effect in amnestic Mild Cognitive Impairment: Associations with hippocampal functional connectivity

Brueggen K*, Kasper E, Dyrba M, Bruno D, Pomara N, Ewers M, Duering M, Buerger K, Teipel S

*** Correspondence:** Katharina Brüggen, katharina.brueggen@dzne.de

1.1 Supplementary Figure

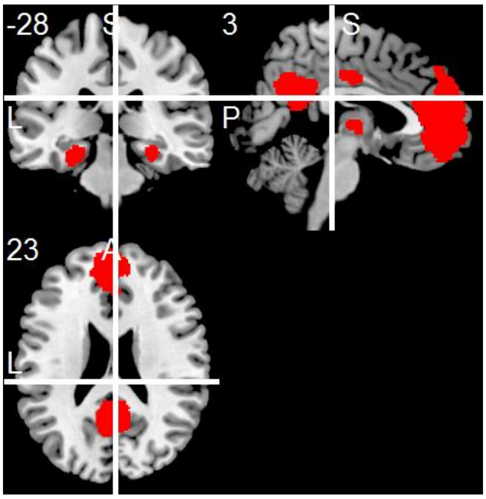

Supplementary Figure 1. Seed regions of the dorsal default mode network (warped to the default IXI template provided by VBM8)
